# Reclaiming selfhood: towards a conceptualisation of the autistic unmasking process

**DOI:** 10.3389/fpsyt.2026.1929738

**Published:** 2026-08-27

**Authors:** Åsa Hedlund, Danielle Unéus, Jonas Wik, Cecilia Ingard, Kajsa Isakson, Melissa H. Black, Tatja Hirvikoski, Hanna Bertilsdotter Rosqvist

**Affiliations:** 1 Faculty of Health and Occupational Studies, Department of Caring Sciences, University of Gävle, Gävle, Sweden; 2 Independent researcher, Visby, Sweden; 3 Independent researcher, Uppsala, Sweden; 4 Department of Social Work, Criminology and Public Health Sciences, University of Gävle, Gävle, Sweden; 5 Independent researcher, Stockholm, Sweden; 6 Department of Community and Clinical Health, School of Allied Health, Human Services & Sport, La Trobe University, Melbourne, Australia; 7 Center of Neurodevelopmental Disorders (KIND), Department of Women’s and Children’s Health, Centre for Psychiatry Research, Karolinska Institutet & Region Stockholm, Stockholm, Sweden; 8 Habilitation & Health, Region Stockholm, Stockholm, Sweden; 9 Faculty of Arts and Social Sciences, Department of Social and Psychological Studies, Karlstad University, Karlstad, Sweden

**Keywords:** autism, identity, masking, neuronormative, unmasking

## Abstract

**Introduction:**

Many autistic people adapt to a neurotypical world through masking, which can lead to exhaustion and harm to mental and physical health. As a result, there is increasingly interest in ‘unmasking’ in ways that improve quality of life and reduce stress. This study analyses qualitative data to explore unmasking among autistic adults.

**Methods:**

This was a neurodivergent-led study. Twenty-nine autistic adults (17 with co-occurring ADHD) were interviewed one to four times as part of a larger project on autistic flourishing. Data related to unmasking were analysed using qualitative content analysis.

**Results:**

Unmasking occurred through a circular process consisting of four categories (1): Identify the need to unmask (2), Gain a deeper understanding of one’s masking (3), Using unmasking strategies, and (4) Managing the consequences of unmasking.

**Conclusion:**

Unmasking can be both challenging and freeing for autistic adults and may be best approached gradually. Supportive environments and professionals who understand masking and its long-term impact and do not encourage masking are important.

## 1 Introduction

### 1.1 Surviving in a neuronormative world: a cost to health?

For many autistic people, navigating a *neuronormative world* (1) requires constant, often exhausting and vigilant, performance. This process, known as autistic masking, involves the conscious and unconscious suppression of aspects regarding one’s behaviour or identity (1, 2) to align with neuronormative expectations and therefore avoid social rejection and misunderstandings in different arenas in childhood and adult life (c.f. 3). In autism research, it is sometimes referred to as *camouflaging* (4, 5), *compensation* (6), *adaptive morphing* (7), or as a form of *impression management* (8). In this article, however, we have chosen to use the term masking because it is an established term within the autistic community in both formal and informal contexts.

Masking is a universal experience, as both neurotypical and autistic people engage in behavioural adaptation and impression management (9, 10). For example, Pryke-Hobbes et al. (11) found that both groups mask in the workplace as a strategy to safeguard against the threat of negative social and employment outcomes. However, autistic masking may be distinguished from neurotypical forms of behavioural adaption or impression management because it is focused on concealing one’s autistic identity and behaviours across different areas of life. This behaviour is often driven by stigma and expectations to conform to neurotypical norms (2). Autistic people have also reported feeling more pressure to mask than neurotypical people, as workplaces are not adapted to autistic needs (11). Autistic people may also mask more frequently than neurotypicals (9) and feel greater pressure to mask autism-specific behaviours such as stimming (10). Autistic people have reported several masking strategies such as suppression of behaviours, performance of a special personality, imitation of others, or scripting responses and phrases (10, 12).

In qualitative research, masking is often described by autistic people as an inevitable part of their daily life (13, 14). One study from the United Kingdom (UK), involving 277 autistic people, found that 79 of them reported masking their autistic characteristics all the time in social situations, to the point that they did not know any other way to behave around others (15). Research has shown that masking is both subjectively experienced (3, 16) and objectively measurable (17) as stressful for the autistic person. It has been associated with poor mental health outcomes such as depression and anxiety (15, 18, 19). It can also have consequences across life domains, such as in the workplace (11) or healthcare (20) where autistic people may be perceived to be “functioning well” according to neuronormative expectations, resulting in a neglect and disregard of support needs.

Masking seems to be a response to both a perceived and actual negative evaluation from others. Indeed, some autistic people have even described being subjected to verbal, emotional, and even physical violence from people who do not accept the autistic person’s neurodivergent behaviour, experiences referred to by some as ‘neurotypical violence’ (21). Masking in social situations often requires preparation, heightened awareness of one’s behaviour during the social interaction, and (negative) self-evaluation afterward, which can be draining (c.f. 22). Because the “mask” is often built out of a necessity for survival and to avoid social exclusion rather than a desire for mimicry (7, 15), it can become internalized, making it difficult for the person to separate their real needs from the neuronormative expectations (10, 23). When masking becomes an automatic mode of self-regulation, it may fundamentally influence the person’s sense of self and being in the world. This chronic state of performance may severely constrain the formation of identity and a sense of self that is distinct from the performed persona (10). A lack of a true sense of self can be serious because it may limit self-awareness, which may place autistic people at greater risk of harmful situations and relationships (24).

While masking is generally negative and linked to a range of poor outcomes, it has also been reported to bring some advantages. For example, it helps to be perceived positively by neurotypical others (25), to be included in neuronormative social contexts (11), and to be employed (3). It can therefore not be said to be all bad or all good. However, even though some masking strategies are adaptive for certain situations, long-term masking often results in exhaustion and long-turn psychological harm (3, 10).

### 1.2 Removing the mask and embracing one’s true self

Since masking has been described as a survival mechanism with largely negative consequences for health, many autistic people report a desire to unmask to a degree that allows for quality of life and reduces the risk of exhaustion and other health problems (11, 26). Unmasking can be described as a process of recognizing and understanding one’s own masking habits to reclaim more authentic (true-to-self) and self-directed ways of being. It involves the deliberate choice to stop suppressing natural neurodivergent responses and to relinquish the adoption of neuronormative alternatives, i.e., be more authentically autistic. Pearson and Rose (2) defined unmasking as *“… the literal representation of autistic persons taking control of their mask and being more authentically themselves” (p 57).* This may include, for example, saying no to expected obligations, feeling free to wear unconventional clothing, no longer adjusting one’s facial expressions based on what one believes other people want (27) or making social interactions more attuned to one´s own pace of cognitive processing (28, 29).

Research on unmasking is an emerging but still limited field and most often appears as a small part of larger studies on masking (e.g., 17). However, the existing research and other sources in the field (e.g., books) points in the same direction: masking is uncomfortable and exhausting, while unmasking also is challenging, albeit desirable (e.g., 5, 16, 27, 29, 30). For example, autistic participants in a study by Rudberg Selin et al. (29) described unmasking as difficult, as masking can feel deeply ingrained and hard to recognize because it has become automatic and habitual. Durben (5) analysed online discussions and found that autistic people wanted to unmask, but that it also entailed negative consequences, such as their close associates perceiving their behaviour as strange, annoying, or unpleasant. Consequently, access to a safe space may be required to unmask (cf. 21, 30). Regarding benefits of unmasking, though requiring further empirical investigation, some have suggested that it may support neurodivergent wellbeing (16, 24, 26, 27, 30), while having a safe space to unmask may support self-esteem, positive autistic identity, mental health, and greater personal authenticity, and may facilitate more genuine relationships (31). Some suggest that unmasking is a process requiring introspective learning and spending time among other neurodivergent people to disentangle which parts of a person’s identity are truly “them” and which are performed for survival (24). It might also involve identifying and stopping specific effortful behaviours, such as forced eye contact or the suppression of stimming, and allowing oneself to be unsociable when the desire to socialize is absent (3). Unmasking is often a complex process involving incremental change over time. Autistic people have described unmasking as involving the gradual process of working through layers of masking across different areas of life. This process can take time and be challenging because masking behaviours may have become deeply ingrained, making it difficult to distinguish between what constitutes masking and what reflects one’s authentic self (30). For some autistic people, the uncertainty associated with unmasking may be perceived as too risky, particularly due to concerns about potential social consequences, such as damaging others’ perceptions of them, jeopardising relationships, or leading to exclusion from social contexts. These concerns may lead autistic people to stop considering or pursuing unmasking (30).

Although there is now emerging evidence on the topic of unmasking, particularly in community literature, there remains a need to empirically investigate unmasking. In this present study, we performed a secondary analysis of a rich qualitative dataset to provide insights into unmasking among autistic adults.

## 2 Methods

### 2.1 Design

This is a neurodivergent-led secondary analysis of qualitative interview data collected as a part of a project exploring autistic flourishing and support services for autistic people. The same dataset has been analysed for another study (28) in the larger project, but with a different aim.

### 2.2 Theoretical standpoint

Our analysis was guided by a neurodiversity approach (32), wherein autism is viewed as part of the natural variation in neural development and functioning. In this way, this study views autism and autistic ways of being as not inherently impaired, but simply different from other (non-autistic) neurotypes way of being.

### 2.3 Positionality

Our team is neurodiverse, including neurotypical and neurodivergent people (autistic and AuDHD, i.e., autism and Attention-Deficit/Hyperactivity Disorder). The broader study in which this analysis was situated was neurodivergent-led, and for the current analysis, neurodivergent researchers primarily undertook data collection, analysis and interpretation of findings.

### 2.4 Community involvement

The broader project involved a co-creation group of autistic and AuDHD community members. The co-creation group was established through existing community networks and consisted of autistic and AuDHD community members. They were invited to contribute based on their interest in research co-creation and their experiential knowledge of autism and neurodivergence. The co-creation group was involved throughout the study, including planning data collection and interpreting findings. They were also included as co-authors in this analysis. A neurodiverse reference group of health care professionals offered broader perspectives and feedback on the project, helping to shape both the interview guide and the interview situation for the broader study in which this data was drawn. The interview guide and procedures were piloted with neurodivergent members of the co-creation, reference, and researcher groups, with the results reported elsewhere (31, 33).

### 2.5 Participants

Participants are the same as those reported elsewhere for the broader project. Participants were required to be 15 years or older. They needed to have an autism diagnosis according to DSM-5 or ICD-10/11 (or DSM-IV equivalent) or self-identify as autistic. They needed to have sufficient Swedish language skills to participate in interviews/focus groups. Among them, 35% were autistic only, and 65% were AuDHD.

### 2.6 Recruitment and data collection

Participants were recruited through advertisements posted on national social media platforms. Potential participants were given access to a short video in which the first author explained the study’s objectives. After consent was given, the first author and each participant collaboratively determined the preferred format and timing of the interview. Participants could choose between a focus group (n=12), an individual spoken interview (n=15), or a written interview (n=3). The interview questions focused on a good autistic life. Examples of questions included, *“What comes to mind when I say a good autistic life?”* and *“What do you do to create a positive feeling within yourself when interacting with others?”*. Even though the interviews were not specifically focused on unmasking, all participants spoke about it. Most participants (n = 19) used the term ‘masking,’ (Swedish: maskering/maskerar eller ‘maska’/’maskar’), while 10 described the experience using other phrases, such as conforming to others’ expectations or the desire to live authentically/as their true selves. The project was designed to include three interview sessions with each participant, spaced about three to four weeks apart, allowing for follow-up sessions and a deeper engagement with the questions. Data collection occurred between March and June 2025. The oral interviews lasted on average 49 minutes.

### 2.7 Data analysis

**Figure 1:**
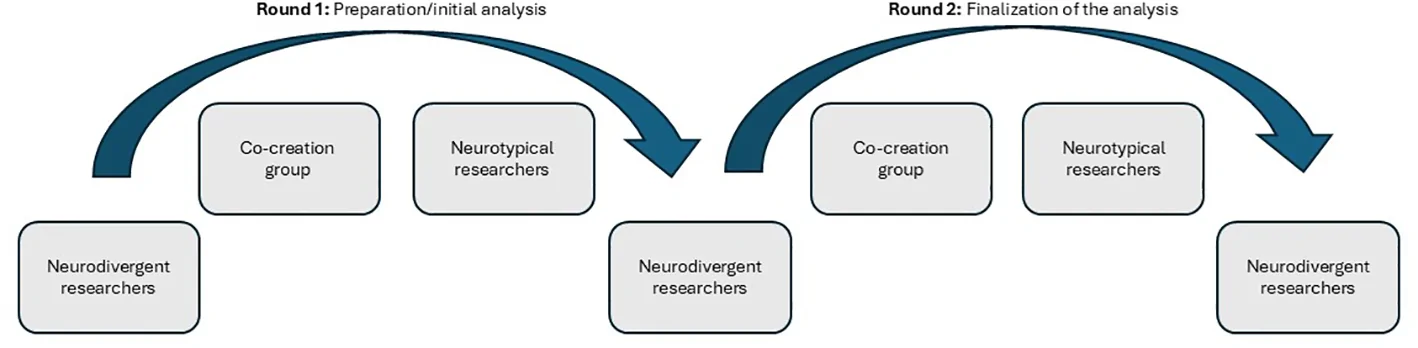
An overview of the order in which different members of the neurodiverse research team review the analytical work in the two rounds of analysis.

The data analysis was conducted inspired by qualitative inductive content analysis according to Graneheim and Lundman (34). This content analysis provided a clear framework to guide the analysis while allowing us to stay close to the interview material. However, some adaptations to the process were necessary to ensure meaningful involvement of all author groups, including allowing additional time for reflection and supporting bottom-up contributions from neurodivergent authors. The research team is neurodiverse, and to ensure a predominantly neurodivergent frame of analysis, the analysis followed two rounds including several steps each (see **Figure 1** for an overview of the rounds):

#### 2.7.1 Round 1: preparation/initial analysis

1. Interviews were transcribed verbatim using Amberscript (https://www.amberscript.com/en/), read through and corrected by first author. All neurodivergent authors read the transcripts
2. All neurodivergent researchers (researchers and the co-creation group) read and commented on the transcripts, supporting initial ideas for possible categories and highlighting aspects that were important to emphasize and retain in the analysis.
3. The interview material was reviewed by the first author to identify meaning units, i.e., parts of interview text ranging from single words to full sentences or paragraphs that were relevant to the study’s aim. These meaning units were organized in a digital table and labelled with participant numbers so they could be traced back to the interview text and the respective participant.
4. Each meaning unit was given a code that captured its central content. Based on similarities and differences between the codes, they were organized into preliminary categories, i.e., *“a group of content that shares a commonality”* (35, p. 5). Unlike Graneheim and Lundman’s linear process, a more iterative approach in the development of codes and categories was used. The formation of preliminary categories began early in the coding process. As more codes emerged, these initial categories were renamed, split, or merged. In this way, coding and categorization proceeded in parallel.
5. The last author reviewed the preliminary analysis and offered critical feedback and suggestions based on neurodivergent lived experience, subject-matter expertise, and research expertise to further strengthen the analysis.
6. The other neurodivergent authors (the co-creation group) reviewed the preliminary analysis and provided critical feedback and suggestions to enhance the credibility and depth of the analysis.
7. The first author continued the analytical development in close collaboration with the last author, integrating the feedback provided.
8. The neurotypical authors reviewed the analysis to date and offered critical feedback and suggestions, drawing on their (clinical) professional perspectives, subject-matter expertise, and research expertise to strengthen the analysis.

#### 2.7.2 Round 2: finalization of the analysis

9. The first and last authors contributed their neurodivergent-informed perspectives while integrating the expertise and perspectives of all authors based on their comments from Round 1 and proposed the final categories.
10. The co-creation group reviewed the proposed final categories and contributed feedback and suggestions to further refine them.
11. The first and last authors further refined the categories and their content in response to the co-creation group’s feedback.
12. The neurotypical authors reviewed the proposed final categories and contributed feedback and suggestions.
13. The first and last authors ensured that the analysis maintained a neurodivergent framework while incorporating the expertise and perspectives of all authors and subsequently finalized the analysis.

## 3 Ethical considerations

The study was approved by the Swedish Ethical Review Authority [Reg. no 2025-01033-01] and was conducted in accordance with relevant legislation and ethical guidelines. All participants provided informed consent prior to the interviews and were given the opportunity to ask questions both before the first interview and throughout the data collection process. Participants were informed that they could withdraw from the study at any time without providing a reason and without any consequences.

## 4 Results

**Figure 2:**
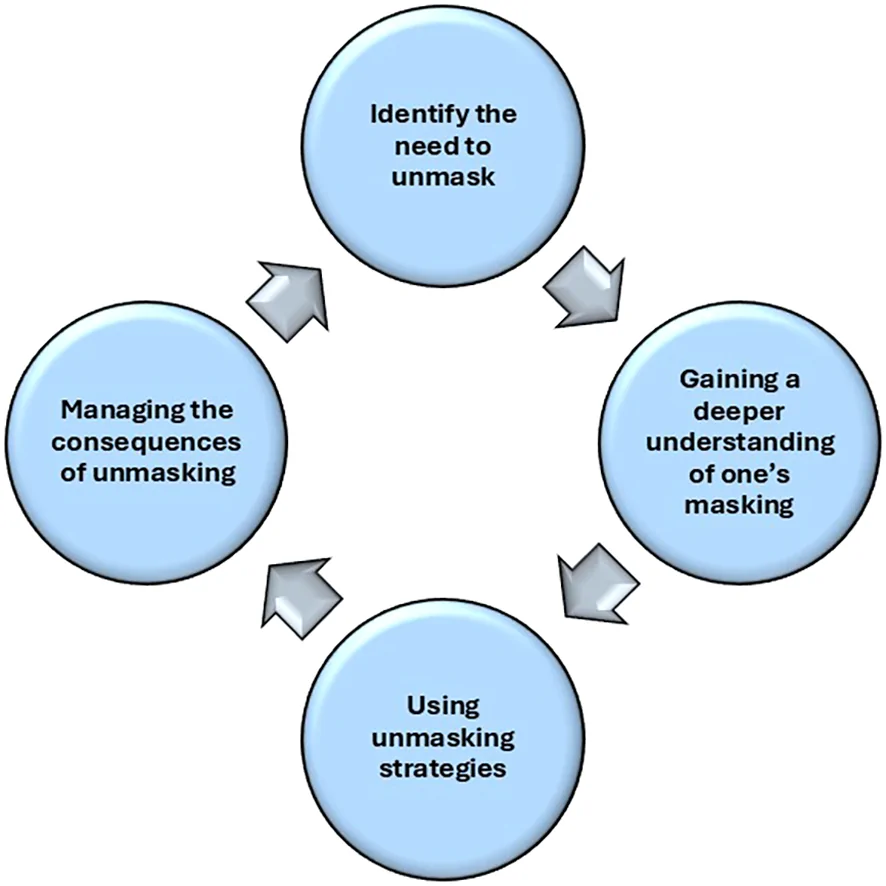
The image shows the four different steps of the unmasking process.

The participants (n=29) were 18–64 years old. Most of them (n=21) identified as women, were born in Sweden (n=26) and had a formal autism diagnosis (n=26). Seventeen reported co-occurring ADHD. The results are presented in four categories, which are interconnected in a circular process (see **Figure 2**). The process begins with identifying the need to unmask, progresses to gaining a deeper understanding of one’s masking behaviours, and then involves using unmasking strategies and managing the consequences of unmasking. Unmasking appears in our study as a relational-contextual construct. It was a process that was described as needing to occur gradually due to perceived relational risks, and as such the process appeared circular (occurring repeatedly across different contexts), rather than linear. Based on our interpretation of their accounts, the participants described varying degrees of unmasking. Some described living almost entirely unmasked lives (i.e., lived a life according to their needs and expressed their natural responses), while others described relatively highly masked lives. The results are supported by participant quotations in italic text, each accompanied by a pseudonymised participant identifier.

### 4.1 Step 1: identify the need to unmask

The first step in the unmasking process was identifying a need to unmask. A majority of participants expressed that they wanted to stop, or significantly reduce, their masking. They described experiencing both psychological and physical distress from masking, reporting that it left them feeling chronically tense in their body and exhausted. One participant described experiencing flare-ups of a physical illness following periods of extensive masking in response to neuronormative expectations, resulting in pain. Another participant described masking as a form of violence against oneself and said that they had had enough:

It’s like doing violence to yourself [ … ] I refuse to adapt! I mean, why can’t they [neurotypicals] adapt? (P24)

The most important reasons for wanting to unmask were the desire to feel better, to save energy, and to be able to view oneself with love rather than with shame or self-criticism. Masking was described as the opposite of self-love, as participants reported forcing themselves into a way of living and being that did not fit their authentic way of functioning:

I’m trying to listen to what I need and accept myself for who I am, instead of, as before, hating myself and being unable to accept my difficulties. (P19)

Participants also described that when they masked, they felt their needs were not taken seriously, leading to the recognition of the need to unmask, “*I felt that I was very close to taking my own life [ … ] the only response I got from them was: call back when you’re feeling worse [ … ] because I usually don’t sound [like I’m unwell] in my voice; they don’t listen to the words!” (P1).* The need to mask led participants to avoid activities that they felt required masking, even though they wanted to engage in the activity in question (e.g., meeting people). Several emphasized that they had not chosen to begin masking voluntarily but had felt compelled to do so from early childhood. This was reported by some to have resulted in low self-awareness and a weak sense of a clear identity:

…for much of my life, I have felt that I don’t even know where I begin and where the masking ends at all. (P15)

Participants also described that the need to unmask grew stronger with age, as they no longer had the energy to mask and to deal with the consequences of masking as they did when they were younger. In adulthood, participants experienced masking as frustrating, since they were rarely given any comprehensible explanation for why neuronormative behaviours (e.g., look people in the eyes) were considered important. Several had also begun to question why they masked, and how important it really was. They questioned the extensive responsibility they had taken on to keep others satisfied and to avoid misunderstandings in communication with others:

It feels like only recently I’ve realized that I can be difficult, that I’m allowed to be difficult, that it doesn’t put me at great risk of something scary to be difficult [ … ] I used to think it was my job to make sure that people always wanted to spend time with me and things like that. (P8)

Despite having a need to unmask, participants reported that there was some resistance to doing so, as it felt uncomfortable to expose their true self, since it was perceived that this could lead to negative criticism. It was also reported by participants to feel uncomfortable simply because they were unaccustomed to it, or because they were afraid of causing others to feel negative emotions:

…I become very anxious and feel very unwell when I think that someone is upset [with me] (P22)

### 4.2 Step 2: gaining a deeper understanding of one’s masking

Once participants became aware of their masking and their desire to unmask, they began reflecting on their masking at a deeper level. Participants reported that they began to interrogate and reflect upon their specific masking behaviours, including when, why, and how they masked. Participants reported that this process was gradual, as many of their masking behaviours were experienced as internalized or subtle.

Participants who had engaged in a deeper level of self-reflection identified several masking behaviours: *bodily* (facial expressions and body language, e.g., maintaining eye contact and sitting ‘properly’ in a chair), *verbal* (things one says), *temporal* (trying to do things at others’ pace, such as by forcing oneself to answer questions quickly in a conversation even if they had not fully processed the content), *cognitive* (pretending to understand when one does not), *being compliant/adaptable* (agreeing to requests without considering personal limits or wants) and *internalized* (mistaking or adopting externally imposed expectations that may not align with personal values or goals). The masking behaviours identified by participants thus included externally observable efforts, but also more internalized processes: One participant described internalized masking:

And it’s also like this now that I’ve discovered that I’m autistic, that maybe what I thought was flourishing wasn’t really as flourishing as I believed, or how should I put it, because it was something I was faking, and which I think led to recurring depressions. So it was some kind of flourishing mask. (P6)

Masking was also described as being either automatic and unconscious or conscious and effortful. When masking was unconscious, participants reported needing to remind themselves to unmask, while conscious masking often involved participants first reading the situation and then deciding what type of masking was needed. One participant expressed:

…I am always sitting there hyperanalyzing their [colleagues’] energy, their tone, their choice of words, so that I can give them what they want. (P22)

Regardless of the specific making behaviour, what they all had in common was that they stemmed from the experience of being unable to relax and be oneself. Generally, participants reported not feeling that they could be themselves with all individuals, except for with those who they shared a household with (e.g., spouses). Some also reported discovering that they masked the most in the presence of neurotypical people, but that they could also mask when interacting with other autistic people because they were unaccustomed to masking around other people, as one described:

I find it really difficult to just stop masking when I’m with other autistic people, and it can easily come across as if I’m not genuine, and it’s true. I’m trying to change that. But it’s so hard, like, to stim, for example, when I’m so used to not doing it. (P4)

Participants reported realizing that they masked to protect themselves from criticism, disapproval, or other negative social consequences. They reported that by behaving in ways that they knew were acceptable and favourable, they could feel safe.

To aid the unmasking process, some participants reported that strengthening their self-awareness was important to understanding their masking, with some describing more a conscious interpretation of their bodily signals, such as whether they were tensing. One participant described:

I’ve started to sometimes think that I can almost feel it in my facial muscles when I’m in a situation where I’m masking a lot, and that I can feel when I come out of it, then it’s as if something physical peels a mask off me when I relax. (P6)

Receiving questions from others about one’s behaviour or feelings, or asking oneself questions through self-reflection, helped participants to further strengthen their self-awareness. Participants reported that it was helpful to reflect upon what they truly wanted and whose needs they were living by. One participant described asking herself whether something felt strange or wrong:

…I think that if there’s something that feels off, something that makes you feel that this is a bit strange, this feels a bit difficult. (P29)

### 4.3 Step 3. using unmasking strategies

Several participants stated that unmasking was challenging, as the masking was deeply ingrained and occurred automatically. Because unmasking was perceived as relationally risky, participants approached it gradually and began with masking behaviours that felt most urgent (i.e., were highly stressful), or were relatively easy to change (for example stopping displaying certain facial expressions). Self-love, for example accepting, respecting, and wanting to protect oneself from suffering, was described as an important motivating factor for unmasking:

Now I respect myself more and try to adapt my life to feel as good as possible, avoiding things that just drain me of energy, for example by being less social and protecting my senses from painful stimuli. (P19)

Participants spoke about different unmasking strategies. Some participants described learning about and adopting other autistic people’s unmasking strategies. Some reported that participating in, or seeing the lives of autistic people who had unmasked, could feel empowering and inspirational. Other strategies included communicating and being open about their needs. For example, expressing to others the need for more processing time during conversations or asking people not to comment on their facial expressions.

Unmasking did not only involve changing specific behaviours in the presence of other people, but could also involve choosing situations in which one was naturally unmasked. For example, several participants had realized that they did not want to spend time with their relatives, but had done so only because it was expected of them. It was also common to begin questioning norms in general and ‘rules’ surrounding what one must and must not do. One participant described how she had come to realize that people have fewer obligations than she had previously believed:

You have no obligation to anything. Obligations are handled at work, or possibly if you have children. Everything else you have the right to say: I don’t want to, and you don’t even need to justify it. It is enough to simply say it. I don’t want to! (P18)

Participants described that they usually began unmasking in “safer” spaces. These spaces were described as those where there was minimal risk (a job interview was an example of a risky space, as a job was at stake). Homes were described as generally safe spaces to unmask; however, unexpected visits could be experienced as stressful due to feelings that their safe space was invaded, especially if they were not completely comfortable being fully unmasked in front of others.

Participants described a range of other unmasking strategies and efforts. Some participants reported preferring to communicate in writing than verbally, spending time at home, choosing a job that aligned with their functioning, seeking out non-critical people, and spending time with Artificial Intelligence (AI) companions instead of human companions. Some parent participants reported that being responsible for another autistic person with similar needs, for example an autistic child, made unmasking easier. For example, participants reported that it was easier to assert the needs of their child/loved one, rather than their own, even if those needs were the same.

### 4.4 Step 4. managing the consequences of unmasking

After unmasking, regardless of the degree, participants had to navigate the relational consequences. Unmasking was not always easy. Several participants emphasized, that unmasking is not solely an individual matter, but requires the environment to support unmasking, making it acceptable to function differently in various parts of society:

[I need] understanding and acceptance of the way I function. That I feel wanted and liked. That I don’t have to struggle so much to choose the right wording when I communicate. (P19)

Unmasking in a social context, even an accepting one, however, was fraught with tensions. Participants reported that it was easy to fall into a pattern of constant apologizing (c.f. 35). Another participant described how she “made fun of” herself or made herself comical to avoid disapproval for her unmasking:

“Yes, yes, I’m weird”. And then I can say it and laugh. (P18)

Some participants emphasized that unmasking required taking into account surroundings, and others’ feelings:

…one should not be a bulldozer. (P12)

Others argued that this, trying not to be a bulldozer, was the wrong focus. They suggested that one should not be afraid to assert one’s needs in ways that might come across as blunt, because it is important that everyone respects each other’s needs. Instead of placing the responsibility for adapting on the autistic person and expecting them to mask, one participant proposed that nobody has to adapt:

Just because I don’t adapt to them doesn’t mean they have to adapt to me. It’s just about being aware of each other. This is what you need, and this is what I need. And then we have to accept that we are different. No one, no one, needs to adapt. (P24)

Participants described that unmasking could lead to feelings of shame (c.f. 36), for example from feeling that they had talked too much. Participants also reported that others could also begin to question how they were feeling, since they no longer arranged their facial expressions in relation to neuronormative expectations (e.g., look constantly happy). If participants were asked that question repeatedly, they reported starting to wonder if there was something to what other people were saying, if they were actually feeling bad after all. They also described how they could be perceived as challenging or provocative when they were simply curious and wanted to understand. Those who dared to pursue interests deemed age-non-neuronormative, described receiving criticism that their interests were too childish.

One participant described experiencing significant fatigue after beginning to unmask, as if their body could finally relax:

I have never been this exhausted before in my life as this year when I realized it [that I am autistic]. Nothing on paper has improved yet, but I think it will. It is a whole life of masking that has to get out of the system somehow. (P6)

However, participants also described improved well-being because of their unmasking. They reported feeling freer to choose things to do and behave in ways that made them feel good, were more able to relax, felt more energetic, and experienced less anxiety. Unmasking went hand in hand with increased self-awareness. A stronger sense of one’s self, that is, knowing who one is and what one needs, was reported by participants to contribute to a feeling of peace. Other positive aspects of unmasking described by participants included having their communication and processing needs met to a greater extent, for example speaking on the phone rather than writing. Several participants described how unmasking led to more accommodation from others than they had expected. One participant described a situation when they began to unmask:

…I was at a café. They had a sandwich there that I had eaten before that was so damn good. [ … ] And when I got there, it wasn’t available. The bread was there, but the toppings were wrong, and I was so disappointed. So I asked: Don’t you have the one with this and that that I had last time? No, they said [ … ] I just said it reflexively: I’m autistic, I don’t like trying new things [ … ]. And then it was like ‘open sesame’ [ … ], no questioning. She was just like: I can make one like that for you. (P18)

The positive consequences of unmasking strengthened the participants motivation to continue their unmasking, bit by bit:

I strive to dare to be myself more and more, to let go of the mask. (P19)

Over time, you can ease up on it more, but I haven’t really gotten to that point at work yet, so I still mask quite a bit there (16).

## 5 Discussion

The results show that unmasking seems to be a circular, relational-contextual process that extends from identifying the need to unmask, through gaining a deeper understanding of one’s masking and applying unmasking strategies, to managing the consequences of unmasking. Since masking needs to be reduced gradually and cautiously, the process seems to be repeated until the person has reached such a low level of masking that it no longer negatively affects their life. The circular unmasking process may be understood as a “unlearning – learning” process where old masking behaviours are “unlearned” and new (unmasked) behaviours are learned.

Parallels may be drawn between the findings of our current study on autistic masking and the experiences and processes of self-disclosure among people living with other concealable stigmatized identities, such as those in the LGBTQIA+ community (c.f. 37). Previous research on other concealable stigmatized identities has proposed a disclosure processes model which is *relational-contextual* and *circular.* Autistic individuals and those with other concealable stigmatized identities have many aspects in common, including stigma and the potential negative impacts for disclosure; however, for many stigmatized identities (e.g., sexual orientation, HIV/AIDs status, and others) masking may involve concealing certain *aspects* of one’s identity, while for autistic individuals masking may be more pervasive and related to behavioural change. Drawing comparisons between our findings however and those of other populations suggest that for autistic individuals, unmasking process must be understood as: 1) relationships between the individual unmasking and the identified audiences, existing in dynamic and fluid environments, 2) as a circular process; as even if some unmasking had occurred, there were always new aspects to unmask/new situations in different environments in which to unmask.

### 5.1 Unmasking in a neuronormative society alongside autistic vulnerability and precarity: a shared responsibility

The results of the study show that masking is complex and nuanced, rather than an “all or nothing” phenomenon. The goal, as expressed by several participants in the current study, may not be total unmasking, but perhaps a less frequent, and more adaptive tool based on one’s own goals and values (c.f. 22). Total unmasking may not ever be realistic given that masking appears to be a universal strategy that all people, regardless of neurotype, feel they need to use at times. For example, to protect oneself or others from embarrassing situations or to obtain intended effects in particular situations (e.g., successfully gaining a job through masking in job interviews). However, autistic masking seems to be extensive and deep-rooted (c.f. 38), going beyond mere impression management by involving the suppression of authentic ways of being and potentially affecting one’s sense of identity, perhaps explaining its particularly negative impact on health and well-being. The findings of this study suggest that while complete elimination of masking may be neither feasible nor necessary, society should still support and encourage opportunities for unmasking, allowing autistic people to express their authentic selves in environments where it is safe to do so. They could also support autistic people in using masking strategically rather than as a destructive survival mechanism, that is, as a tool in situations where the person wants to mask, for example to avoid missing out on an important employment opportunity (c.f. 39). Social skills support could shift its focus from training autistic people to act according to neuronormative expectations, and instead focus on communicating needs, advocating for oneself, understanding social norms, and developing self-awareness through neurodivergent-affirmative introspection analyses (as illustrated by the first two steps of the unmasking process).

Within the context of conversations on masking and unmasking, it is important to acknowledge how challenging forming an autistic self-awareness in a neuronormative world can be. The findings of the present study indicated that participants sometimes masked unconsciously even to themselves, believing they were doing well when they were not. According to Miller et al. (10), prolonged and pervasive masking can result in a fractured identity and confusion regarding one’s true self. What is unique about the present findings is that this confusion about oneself is not always consciously experienced by the autistic person, until they begin to *analyse* their (neuronormative-adapted) masking behaviours. This can be interpreted as a particular kind of introspective analyses, whereby individuals gradually, over time, gain an awareness and understanding of their behaviours, moving away from neuronormative-adapted strategies and toward more neurodivergent-affirming (unmasking) behaviours. This has important implications for formal support providers, particularly in the context of person-centred care and participation. If autistic people lack sufficient self-awareness, it becomes difficult for them to accurately communicate their needs in response to direct questions. As a result, person-centred approaches may appear to be effective on the surface, while in reality failing to fully capture and address the person’s actual experiences and needs. The fact that support providers may unintentionally reinforce masking behaviour by underestimating the abilities (e.g., capability, motivation) of those who do not mask is another barrier to unmasking (40), that needs to be eliminated by increased knowledge about autistic ways of being.

Unmasking in predominantly neuronormative spaces put demands on already vulnerable people to take responsibility for their masking, including risks of secondary victimization (i.e., “being punished again” for being more open) as has been explored in the context of sexual victimization following disclosure of sexual assault (c.f. 41). This becomes particularly problematic when unmasking behaviours are involuntary, such as in autistic burnout, where the person lacks the energy to mask (16), as they may then become caught in a negative cycle of exhaustion and secondary victimization, such as “social punishment”. On the other hand, the results from the present study showed that when one dares to unmask and express one’s needs, it can be received by others more positively than one had anticipated beforehand and hereby support healing from trauma and neuroableism. This illustrates presence of autistic vulnerability and precarity, what Dryden and Catala (1) has referred to as *“openness vulnerability”*, in the unmasking process. It means being able to be open about one’s authentic self without being harmed. Against this background, it is important to have an open dialogue about needs and ways of functioning among both neurotypical and neurodivergent people, in different areas of society, so that discussing these matters becomes more natural and any potential prejudices can be addressed.

The results indicate that unmasking can sometimes lead to negative social consequences, such as criticism or judgment from others, which may evoke feelings of shame or stigma in autistic people (c.f. 36, 42). These experiences highlight that unmasking is not without risk (c.f. *“harm vulnerability”* in 1), and navigating the social repercussions can be emotionally challenging. Research by Botha and Frost (43) involving 111 autistic adults also found that “outness” (i.e., disclosing one’s autism to others) was associated with lower psychological well-being. The authors suggest that this may be because outness may expose autistic people to greater discrimination from others, in contrast to outness among sexual minorities, where it has been shown to have potentially beneficial and healing effects (44). This implies that the social environment needs to support unmasking so that participants’ journey toward a less masked life does not become harmful to them.

Participants reported joking about themselves, or repeatedly apologizing for their non-neuronormative behaviour, when their neurodivergent self-became visible to others, as a way to protect against disapproval. This behaviour can be understood as an expression of internalized neuroableism, which is the internalization of society’s negative views of autistic behaviour (45). By making themselves the subject of ridicule, they attempt to soften potential criticism, but at the same time, it reinforces the message that autistic ways of being are not accepted. This can hinder society’s ability to value and accept autistic behaviour on its own terms, leading to sustained stigmatization. Research has shown that when autistic people are in a neurodiversity-affirmative environment, they develop a more positive autistic self-awareness, which in turn is associated with better health and well-being (46). It is therefore important to have access to neurodiversity-affirmative environments, both in family life and in formal contexts. Even though a neurodiversity-affirmative environment is important, a central aspect of unmasking according to the present findings is to stop invoking people-pleasing behaviours or constantly trying to be liked by others (one participant described that they used to think it was their job to make people always want to spend time with them): This can be understood as fawning, that is, to surrender one’s boundaries, over-accommodating, and submitting to people one perceives as having harmed them (47). The autistic person’s self-imposed responsibility for creating a positive and supportive social environment in the unmasking process therefore needs to be carefully explored in shadows of fawning. At the stage of living an almost entirely unmasked life, remaining masking behaviours may be used sporadically and strategically, rather than as a daily compensation for the surrounding environment’s lack of willingness to be neurodiversity-affirmative.

### 5.2 Supporting self-awareness

Several masking behaviours were discussed in the results, partly in line with previous research (e.g., 13). However, in the present study, we found masking areas (the body, temporal, etc.), whereas in previous research (e.g., 13) descriptions of masking are limited to what the person does (i.e., which strategies are applied). Furthermore, both “automatic” and “non-automatic” masking strategies were described by the participants in the present study, in line with Pearson and Rose (2), who described masking in autistic people as both conscious and unconscious. The presence of both types of masking has implications for the unmasking process. As described in the results, some masking strategies are difficult to identify by the person themselves, whereas more deliberate strategies can be identified and targeted more easily. It is reasonable to assume that unconscious masking strategies can continue to exert stress and increase cognitive load (leaving less cognitive capacity for other processes, c.f. 48) even when conscious masking is reduced, highlighting the need for an ongoing and prolonged journey of neurodiversity-affirmative personal reflection. Supporters, such as family members, and clinicians, have an important role in facilitating this process by providing understanding, safe spaces, and encouragement for exploring autistic self-awareness through introspective analysis (49).

Previous descriptions of the unmasking process have focused primarily on self-awareness of one’s *true self* (25, 50). However, the results of the present study suggest that unmasking also explicitly involves the need to identify (analyse) *one’s masking.* Identifying both one’s conscious and unconscious masking strategies appears to be a necessary step to be able to live a more authentic life. This suggests that the concept of autistic self-awareness (49) should be understood more broadly to include not only insight into an authentic autistic way of being (51) but also recognition of the integrated strategies and adaptations one has developed in response to neuronormative expectations and survival in a neuronormative world. Peer support through the autistic community may be an important resource here. Partly for neurodiversity-affirmative recognition and sharing tips, and partly because autistic people may experience a reduced need to mask when interacting with other autistic people (52, 53), which can potentially create a more relaxed environment but may also function as a caring space, supporting personal growth from internalised neuroableism and trauma (c.f. 36). The results also indicated that participants could be inspired by other autistic people regarding unmasking strategies they could use. While this may provide valuable support, it is important to consider that adopting others’ strategies before developing sufficient self-awareness may risk creating another form of masking. For example, if autistic people adopt behaviours or strategies based on how other autistic people unmask, without considering whether these strategies reflect their own needs and preferences, they may begin to adopt ways of acting that do not correspond to their authentic way of expressing themselves. It is also important to note that autistic people are diverse and may not always be compatible with one another (54); therefore, relationships between autistic people cannot always be assumed to be friction-free.

While the topic of the current study was unmasking, the results reinforce those of previous studies on masking, showing that autistic people mainly experience masking as stressful and negative (e.g., 3, 11, 18, 55, 56), highlighting the need for unmasking. There is also an important gender aspect to this. Healthcare systems tend to identify autism in girls and women later than in men (57), which means they may, for a longer period, lack the tools to understand themselves and, consequently, to develop effective strategies in relation to masking and unmasking. Society and healthcare therefore need to continue their efforts towards earlier identification of autistic girls and women, as well as provide support for the subsequent process of developing self-awareness.

## 6 Limitations and future research

This study has several limitations that should be highlighted. First, most participants were women born in Sweden, which limits the transferability of the findings to men and people with other gender identities, as well as to people born and residing in other countries. As none of the participants were non-verbal or had intellectual disability limiting transferability to these populations. Another important limitation is that the study was not specifically designed to investigate the phenomenon of unmasking, which may have resulted in some important aspects of unmasking not being captured. Future research would benefit from conceptualising unmasking, for example by validating the circular model or exploring the applicability of similar models (i.e., the disclosure processes model (37) to autistic people. It is also important to note that many participants in the present study had co-occurring ADHD (referred to as AuDHD when occurring alongside autism). Although masking has also been documented in ADHD people (58), the research on masking and unmasking in ADHD and AuDHD is less developed than the corresponding literature on autism. Consequently, the findings of the present study may also reflect experiences of AuDHD masking, although we did not distinguish between autistic masking and AuDHD masking. Further research is needed to better understand the similarities and differences between these forms of masking. In addition, given the conceptual overlap between masking and other related constructs such as compensation, impression management, and camouflaging within both neurotypical and other populations (e.g., social anxiety), research is needed to disentangle the shared and distinct experiences, mechanisms, and processes.

## 7 Conclusion

Overall, the study suggests that unmasking is an important process for autistic people but one that needs to occur gradually and iteratively. It involves multiple challenges, and autistic people may experience precarity and vulnerability, but also a newfound sense of freedom. From participant accounts, it appears to be a circular process, beginning with identifying a need to unmask, gaining a deeper understanding of one’s masking, followed by the use of different unmasking strategies, and managing the negative and positive consequences that unmasking entails. The findings further indicate that it is important for the surrounding environment to support unmasking and not perceive it as a threat to neurotypical ways of being. Finally, formal support providers need to recognize the challenges associated with forming an autistic self-awareness and a positive autistic identity in a neuronormative world, among them long-term masking (that has influenced the experience of the self) in autistic adults when seeking to involve them in support and treatment.

## Data Availability

The datasets presented in this article are not readily available because they contain sensitive personal data, and ethical and legal restrictions prohibit data sharing. Requests to access the datasets should be directed to asahed@hig.se.

## References

[1] Dryden J Catala A . Shifting the vulnerability economy: Autism and unmasking. Hypatia. (2026), 1–19. doi: 10.1017/hyp.2026.10080 41292463

[2] Pearson A Rose K . Autistic masking: Understanding identity management and the role of stigma. In: Pavilion Publishing and Media Ltd. Sea, West Sussex: Pavilion Publishing and Media Ltd (2023).

[3] Field SL Williams MO Jones CRG Fox JRE . A meta-ethnography of autistic people's experiences of social camouflaging and its relationship with mental health. Autism. (2024) 28:1328–43. doi: 10.1177/13623613231223036 38197398 PMC11134975

[4] Arnold WM Bitsika V Sharpley CF . Camouflaging and autism: Conceptualisation and methodological issues. Autism. (2026) 30(5):1131–46. doi: 10.1177/13623613261420085 41721649 PMC13087159

[5] Durben DL . Understanding autistic camouflaging: The use of online community discussions and stigmatized identity research. Neurodiversity. (2024) 2. doi: 10.1177/27546330241266726

[6] Livingston LA Colvert E Social Relationships Study Team . Good social skills despite poor theory of mind: Exploring compensation in autism spectrum disorder. J Child Psychol Psychiatry. (2019) 60:102–10. doi: 10.1111/jcpp.12886 29582425 PMC6334505

[7] Lawson WB . Adaptive morphing and coping with social threat in autism: An autistic perspective. J Intellect Disabil Diagn Treat. (2020) 8:519–26. doi: 10.6000/2292-2598.2020.08.03.29 39637746

[8] Khudiakova V Alexandrovsky M Ai W Lai MC . What we know and do not know about camouflaging, impression management, and mental health and wellbeing in autistic people. Autism Res. (2025) 18:273–80. doi: 10.1002/aur.3299 39719862 PMC11826008

[9] van der Putten WJ Mol AJJ Groenman AP Radhoe TA Torenvliet C van Rentergem JAA . Is camouflaging unique for autism? A comparison of camouflaging between adults with autism and ADHD. Autism Res. (2024) 17:812–23. doi: 10.1002/aur.3099 38323512

[10] Miller D Rees J Pearson A . Masking is life”: Experiences of masking in autistic and nonautistic adults. Autism Adulthood. (2021) 3:330–8. doi: 10.1089/aut.2020.0083 36601640 PMC8992921

[11] Pryke-Hobbes A Davies J Heasman B Livesey A Walker A Pellicano E . The workplace masking experiences of autistic, non-autistic neurodivergent and neurotypical adults in the UK. PloS One. (2023) 18:e0290001. doi: 10.1371/journal.pone.0290001 37672533 PMC10482295

[12] Nel J Spedding M Malcolm-Smith S . Consolidating a framework of autistic camouflaging strategies: An integrative systematic review. Autism. (2025) 29:2379–94. doi: 10.1177/13623613251335472 40448317 PMC12417612

[13] Pollock A Krupka Z . Late bloomers: Exploring the emotional landscape of Australian women’s experiences of a late autism diagnosis. Autism. (2026) 30:426–38. doi: 10.1177/13623613251386983 41169026

[14] García-Simón MI Navarro-Jiménez EDM Martínez-Ortigosa A Ropero-Padilla C Román P Rodríguez-Arrastia M . Experiences of females with late diagnosis of autism: Descriptive qualitative study. Nurs Res. (2025) 74:288–93. doi: 10.1097/NNR.0000000000000825 40232942

[15] Bradley L Shaw R Baron-Cohen S Cassidy S . Autistic adults' experiences of camouflaging and its perceived impact on mental health. Autism Adulthood. (2021) 3:320–9. doi: 10.1089/aut.2020.0071 36601637 PMC8992917

[16] Osborne C Barker J Cole A Russell R . Autistic people’s experience of camouflaging and autistic burnout in relation to one another: A systematic review. Res Neurodivers. (2026) 2:100025. doi: 10.1016/j.rin.2026.100025 42574925

[17] Zubizarreta SC Isaksson J Faresjö Å Faresjö T Carracedo A Prieto MF . The impact of camouflaging autistic traits on psychological and physiological stress: A co-twin control study. Mol Autism. (2025) 16:59. doi: 10.1186/s13229-025-00695-9 41299653 PMC12659362

[18] Oshima F Takahashi T Tamura M Guan S Seto M Hull L . The association between social camouflage and mental health among autistic people in Japan and the UK: A cross-cultural study. Mol Autism. (2024) 15:1. doi: 10.1186/s13229-023-00579-w 38178255 PMC10768303

[19] Ross A Grove R McAloon J . The relationship between camouflaging and mental health in autistic children and adolescents. Autism Res. (2023) 16:190–9. doi: 10.1002/aur.2859 36416274

[20] Grant A Turner S Shaw SCK Williams K Morgan H Ellis R . I am afraid of being treated badly if I show it”: A cross-sectional study of healthcare accessibility and Autism Health Passports among UK autistic adults. PloS One. (2024) 19:e0303873. doi: 10.1371/journal.pone.0303873 38809913 PMC11135756

[21] Radulski EM . Conceptualising autistic masking, camouflaging, and neurotypical privilege: Towards a minority group model of neurodiversity. Hum Dev. (2022) 66:113–27. doi: 10.1159/000524122

[22] Black MH Clarke PJF Deane E Smith D Wiltshire G Yates E . That impending dread sort of feeling": Experiences of social interaction from the perspectives of autistic adults. Res Autism Spectr Disord. (2023) 101:102090. doi: 10.1016/j.rasd.2022.102090 42574925

[23] Hennekam S Walkowiak E Szulc J . How neurodivergent workers use and make sense of assistive technologies: Implications for the AMO model and digital masking. New Technol Work Employ. (2026):e70025. doi: 10.1111/ntwe.70025 40046247

[24] Mutti-Driscoll C . Just be yourself” isn’t so simple: How to stop masking & live authentically. In: Hallowell Todaro Adhd & Behavioral Health Center. Harmony, NY (2025). Available online at: https://www.hallowelltodaro.com/ (Accessed June 30, 2026).

[25] Klein J Krahn R Howe S Lewis J McMorris C Macoun S . A systematic review of social camouflaging in autistic adults and youth: Implications and theory. Dev Psychopathol. (2025) 37:1320–34. doi: 10.1017/S0954579424001159 39370528

[26] Raymaker DM Teo AR Steckler NA Lentz B Scharer M Delos Santos A . Having all of your internal resources exhausted beyond measure and being left with no clean-up crew”: Defining autistic burnout. Autism Adulthood. (2020) 2:132–43. doi: 10.1089/aut.2019.0079 32851204 PMC7313636

[27] Price D . Unmasking Autism: The Power of Embracing Our Hidden Neurodiversity. London: Octopus Publishing Group (2022).

[28] Hedlund Å Lindström T Bölte S Black MH Hirvikoski T Isakson K . Defining autistic flourishing: Exploring lived experiences through a neurodivergent lens. Autism Adulthood. (2026) 0(0). doi: 10.1177/25739581261462070

[29] Selin TR Unéus D Knudsen S . (2026). “ Chasing shadows”: Understanding personal data externalization and self-tracking for neurodivergent individuals”, in: Proceedings of the 2026 CHI Conference on Human Factors in Computing Systems (New York, NY: Association for Computing Machinery). doi: 10.1145/3772318.3794641

[30] Durben DL . Unmasking: A mixed-methods study of autistic college students' efforts to stop masking/camouflaging. J Autism Dev Disord. (2026). doi: 10.1007/s10803-026-07409-x 42435102

[31] Hedlund Å Eriksson Wester M Edenvik P Ingard C Isakson K Kron Sabel L . A good autistic life: An autistic-led conceptualization of autistic flourishing through autistic women’s lived experiences. Front Sociol. (2025) 10:1611803. doi: 10.3389/fsoc.2025.1611803 41069404 PMC12506894

[32] Dwyer P . The neurodiversity approach(es): What are they and what do they mean for researchers? Hum Dev. (2022) 66:73–92. doi: 10.1159/000523723 36158596 PMC9261839

[33] Hedlund Å Eriksson Wester M Edenvik P Ingard C Isakson K Kron Sabel L . Methodological insights from the inside: Developing autistic-friendly interviewing in a group interview space. Front Psychiatry. (2026) 16:1659580. doi: 10.3389/fpsyt.2025.1659580 41675666 PMC12886361

[34] Graneheim UH Lundman B . Qualitative content analysis in nursing research: Concepts, procedures and measures to achieve trustworthiness. Nurse Educ Today. (2004) 24:105–12. doi: 10.1016/j.nedt.2003.10.001 14769454

[35] Williams GL Ellis R Holloway W Caemawr S Craine M Williams K . ‘Building our own house’ as an insider-only community-partnered participatory research council: Co-creating a safe space for autistic knowledge production. Autism. (2025) 29:2205–15. doi: 10.1177/13623613241253014 38757637 PMC12332215

[36] Doherty M . Autistic shame: Reflections on an under-recognised determinant of autistic mental health (2026). Available online at: https://www.researchgate.net/publication/403574843_Autistic_Shame_Reflections_on_an_Under-Recognised_Determinant_of_Autistic_Mental_Health (Accessed June 30, 2026).

[37] Chaudoir SR Fisher JD . The disclosure processes model: Understanding disclosure decision making and postdisclosure outcomes among people living with a concealable stigmatized identity. psychol Bull. (2010) 136:236–56. doi: 10.1037/a0018193 20192562 PMC2922991

[38] Burns TM Botha M . Who am I: The balance between masking and identity. Autism Adulthood. (2025) 0(0). doi: 10.1177/25739581251365476

[39] Cage E Troxell-Whitman Z . Understanding the reasons, contexts and costs of camouflaging for autistic adults. J Autism Dev Disord. (2019) 49:1899–911. doi: 10.1007/s10803-018-03878-x 30627892 PMC6483965

[40] Dalmo MOV . How support systems interpret and reinforce autistic masking: Introducing the concept of ‘visibility logic.’. Disabil Soc. (2026) 41:1910–33. doi: 10.1080/09687599.2026.2659101 37339054

[41] Jackson MA Valentine SE Woodward EN Pantalone DW . Secondary victimization of sexual minority men following disclosure of sexual assault: “Victimizing me all over again…. Sex Res Soc Policy. (2017) 14:275–88. doi: 10.1007/s13178-016-0249-6

[42] Morris IF Sykes JR Paulus ER Dameh A Razzaque A van Esch L . Beyond self-regulation: Autistic experiences and perceptions of stimming. Neurodiversity. (2025) 3. doi: 10.1177/27546330241311096

[43] Botha M Frost DM . Extending the minority stress model to understand mental health problems experienced by the autistic population. Soc Ment Health. (2020) 10:20–34. doi: 10.1177/2156869318804297

[44] Legate N Ryan RM Rogge RD . Daily autonomy support and sexual identity disclosure predicts daily mental and physical health outcomes. Pers Soc Psychol Bull. (2017) 43:860–73. doi: 10.1177/0146167217700399 28903670

[45] Jóhannsdóttir Á Egilson SÞ Haraldsdóttir F . Implications of internalised ableism for the health and wellbeing of disabled young people. Sociol Health Illn. (2022) 44:360–76. doi: 10.1111/1467-9566.13425 35034362 PMC9304167

[46] Davies J Cooper K Killick E Sam E Healy M Thompson G . Autistic identity: A systematic review of quantitative research. Autism Res. (2024) 17:874–97. doi: 10.1002/aur.3105 38334318

[47] Brown E . Fawning, masking, and working as an intimacy professional on the autism spectrum. J Consent Based Perform. (2023) 2:74–82. doi: 10.46787/jcbp.v2i2.3862

[48] Clark C Kimmons R . Cognitive load theory. In: Edtechnica: The Open Encyclopedia of Educational Technology. EdTech Books (2023). p. 109–15. doi: 10.59668/371.12980

[49] Bertilsdotter Rosqvist H Hultman L Hallqvist J . Knowing and accepting oneself: Exploring possibilities of self-awareness among working autistic young adults. Autism. (2023) 27:1417–25. doi: 10.1177/13623613221137428 36409056 PMC10291373

[50] Neff MA . Reflections on unmasking. In: Neurodivergent Insights. Neurodivergent Insights (2021). Available online at: https://neurodivergentinsights.com/reflections-on-unmasking/ (Accessed June 30, 2026).

[51] Sinclair J . Don’t mourn for us. In: Our Voice, vol. 1. (1993). Available online at: https://www.autreat.com/dont_mourn.html (Accessed June 30, 2026).

[52] Scheeren AM Nieuwenhuis S Crane L Roke Y Begeer S . Masking, social context and perceived stress in autistic adults: an ecological momentary assessment study. Autism. (2025) 29:3002–13. doi: 10.1177/13623613251353358 40635406 PMC12618727

[53] Khudiakova V Levy X Sowden-Carvalho S Surtees ADR . A qualitative exploration of autistic people’s experiences of camouflaging across different social contexts. Autism Adulthood. (2025) 0(0). doi: 10.1089/aut.2024.0288 36698420

[54] Minnell H Waddington H Jordan P Noble B Bowden CJ . I accept them, they accept me, we enjoy our time together": Autistic adults' preferences and perceptions of relationships with other autistic people. Autism. (2026) 30(8):2000–14. doi: 10.1177/13623613261451898 42349898 PMC13392157

[55] Evans JA Krumrei-Mancuso EJ Rouse SV . What you are hiding could be hurting you: Autistic masking in relation to mental health, interpersonal trauma, authenticity, and self-esteem. Autism Adulthood. (2024) 6:229–40. doi: 10.1089/aut.2022.0115 39139513 PMC11317797

[56] Dabbs CR Hutchins CH Baird R Scaer AJ Kosanovich SE Spitler-Nigh B . Unmasking bias: Autistic perspectives in mental health training. Autism Adulthood. (2026) 8(3):468–81. doi: 10.1089/aut.2024.0210 36698420

[57] Fyfe C Winell H Dougherty J Gutmann DH Kolevzon A Marrus N . Time trends in the male to female ratio for autism incidence: population based, prospectively collected, birth cohort study. BMJ. (2026) 392:e084164. doi: 10.1136/bmj-2025-084164 41638711 PMC12961995

[58] Maeda C Knouse LE Takeda K Masudomi C Takahashi E Katsuragawa T . A narrative review of stigma and masking in ADHD: insights from English-language research and the Japanese cultural context. Front Psychol. (2026) 17:1807337. doi: 10.3389/fpsyg.2026.1807337 41987980 PMC13076299

